# ePhyLi digital tools improve student-teachers' physical literacy knowledge: evaluation of usability, user experience, and learning outcomes

**DOI:** 10.3389/fspor.2026.1875835

**Published:** 2026-07-22

**Authors:** Antonino Mulè, Manolis Adamakis, Anna Rouvi, Jana Vašíčková, Nigel Green, Olia Tsivitanidou, Nicos Kasenides, George Kalmpourtzis, Efstathios Christodoulides, Attilio Carraro

**Affiliations:** 1 Faculty of Education, Free University of Bozen-Bolzano, Bozen-Bolzano, Italy; 2 School of Physical Education and Sport Science, National and Kapodistrian University of Athens, Athens, Greece; 3 GrantXpert Consulting Limited, Nicosia, Cyprus; 4 Faculty of Physical Culture, Palacký University Olomouc, Olomouc, Czechia; 5 European Physical Education Association (EUPEA), Esch-sur-Alzette, Luxembourg; 6 International Physical Literacy Association, Wigan, United Kingdom; 7 School of Science, University of Lancashire (UCLan Cyprus), Larnaka, Cyprus; 8 Inquiry Fuse, Nicosia, Cyprus; 9 Infinitivity Design Labs, Laussonne, France

**Keywords:** digital technologies, digitalization, e-book, e-platform, mobile app, physical education, physical literacy, teacher training

## Abstract

**Introduction:**

Physical Literacy (PL) is widely recognized as an essential focus for promoting active lifestyles and lifelong healthy habits. Equipping teachers and student-teachers, particularly those in primary and physical education, with significant PL-related knowledge is essential for fostering lifelong healthy behaviors from childhood. This study aimed to present the ePhyLi Erasmus + Sport project contents and to evaluate the effectiveness, usability, and user satisfaction of educational materials developed within the project (e-book, mobile app, and e-platform).

**Methods:**

Eighty-six student-teachers (38 males; aged 19–26) were invited to participate in the study. To investigate the tools' adaptability to teaching strategies commonly employed in higher education, three models were used: curricular integration, autonomous study, and mixed approach. PL knowledge improvement was assessed using a 20-item questionnaire administered pre- and post-intervention. User experience was comprehensively evaluated using the User Experience Questionnaire short version, the System Usability Scale, the Gameful Experience Questionnaire, and the App Usability Evaluation Questionnaire. Self-perceived usefulness, understanding, and gamification of the e-platform and the app were evaluated by administering nine single-ended questions.

**Results:**

Findings showed that the ePhyLi tools significantly improved participants' PL knowledge, particularly when the e-book was integrated into weekly lectures, alongside the use of the e-platform and app (curricular integration model). User experience was moderately positive, while system usability showed marginal issues. The app usability questionnaire showed a generally positive result, and the gamification elements were perceived as highly engaging and motivating. Participants reported strong self-perceived learning and increased confidence in applying PL concepts, rating the tools favorably for overall educational utility. However, primary school student-teachers reported significantly lower system usability, overall satisfaction, and playfulness when using the app compared to Sport Science students.

**Conclusion:**

Digital innovation and gamification can be valuable tools for improving student-teachers' training in promoting physical literacy. Although the ePhyLi tools were effective in increasing knowledge, some areas need future attention. Future studies are needed to improve the amount of evidence that supports these results and to evaluate whether the increased knowledge gained through ePhyLi tools translates into improved teaching practices in schools.

## 1 Introduction

Physical literacy (PL) can be described as the motivation, confidence, physical competence, knowledge and understanding to value and take responsibility for engagement in physical activities for life (1). In recent years, the concept of PL has emerged as a key point in international policy and practice, recognized for its relevance in fostering active individuals and societies (2). It can be considered an individual's journey. Cairney et al. (3) emphasized that PL is a lifelong developmental process based on an individual's relationship with movement and physical activity (PA). In their model, the authors showed that positive PA experiences influence PL, which in turn influences participation in PA and health outcomes later in life. A disposition towards engaging in movement and PA throughout life is proven to help prevent and manage noncommunicable diseases such as heart disease, hypertension, stroke, diabetes and several cancers, as well as improve mental health, quality of life and well-being (2).

School settings play a crucial role in fostering PL and PA. As a core concept in Physical Education (PE), PL promotes lifelong engagement by consolidating the physical, emotional, cognitive, and social skills necessary for youth development. According to UNESCO, PL is one of the pillars of the Quality Physical Education, promoting the enhancement of children and adolescents' motivation, confidence, physical competence, knowledge and understanding that are crucial for nurturing healthy, capable and active citizens (4). The GloPL Consortium (5) highlights the importance of equipping future educators with the knowledge and skills to promote PL, as PE and primary school teachers directly impact students' lifelong PA and well-being. Evidence highlighted a clear need to strengthen professional development to integrate PL more effectively in practice (6). Although some countries have already integrated PL objectives into their national educational curricula (7–9), these curricula only moderately met UNESCO's and WHO's claims for policy and curricular alignment (9). Notable gaps remain in areas such as student-centered teaching, assessment methods, and the acknowledgement of individuals' life journeys (6).

Universities can play a critical role in this process by providing purposefully designed education curricula that embed PL into initial teacher training. These programs can equip the next generation of teachers with a robust understanding of the PL concept, while providing the appropriate teaching experiences and skills needed to act as a role model for their future pupils. However, in this context, a significant gap remains in the literature, especially regarding how the digital transformation of higher education curricula and the integration of gamified learning approaches can effectively support initial teacher education in enhancing PL knowledge. To address this specific need, the ePhyLi project was developed. Founded by the Erasmus + Sport program, the project, primarily dedicated to university students in PE and primary education, aimed to: (a) assess university students' understanding of PL and how this influences their personal engagement in PA; (b) contribute to the digital transformation of university curricula by developing open-access digital educational tools for promoting PL within university curricula and training programs, using a gamified digital learning approach; (c) increase students' knowledge and understanding of PL and educate them on the importance of a healthy lifestyle; and (d) prepare future teachers to promote PL to their students, as part of their future professional practice. The present paper aims to inform readers about the ePhyLi project, to evaluate the effectiveness of the tools in enhancing knowledge and understanding of PL, and to assess users’ experiences with tools usability and satisfaction. Specifically, the study addressed the following Research Questions (RQ):

- RQ1: To what extent do the ePhyLi digital tools improve university student-teachers' knowledge of PL?
- RQ2: How does the mode of implementation (curricular integration, autonomous study, or mixed approach) influence learning outcomes?
- RQ3: How do users evaluate the usability, user experience, and educational value of the ePhyLi e-platform and mobile application?

## 2 Materials and methods

### 2.1 Participants

Eighty-six student-teachers (38 males and 48 females; aged 19–26) gave their consent to take part in the study. The sample was composed by 29 student-teachers currently in their 3rd and 4th year enrolled in the BSc (Hons) Sport and Exercise Science program at the University of Central Lancashire—Cyprus (UCLan Cyprus), 25 student-teachers currently in their 3rd or 4th year of the BSc program in Sports Science and PE at the European University Cyprus, and 32 student-teachers attending the 2nd year of the Primary School Teachers single-cycle master's degree at the Faculty of Education of the Free University of Bozen-Bolzano. The study was approved by the Cyprus National Bioethics Committee (*ΕΕΒΚ ΕΠ* 2023.01.34). Written consent forms were obtained from all the participants.

### 2.2 Study design

According to the three universities, participants were divided into three groups: UCLan Cyprus (G1), European University Cyprus (G2), and Free University of Bozen-Bolzano (G3). During the piloting activities, participants were first introduced to the ePhyLi project and completed the pre-intervention questionnaire. To investigate the adaptability of the ePhyLi tools to different teaching strategies commonly employed in higher education, three implementation models were used:

- Curricular integration: A curricular integration of the e-book into weekly lectures throughout a semester was provided to G1. After completing the weekly lecture series, which incorporated flipped-classroom methods, participants interacted with the e-platform and the three interactive learning modules during a dedicated workshop. Students were then guided in using the mobile application. On the same day, participants filled in the post-intervention questionnaire.
- Autonomous study: After guiding G2 through the e-book contents and introducing participants to both the e-platform, the three interactive learning modules, and the mobile app, students were encouraged to explore the educational resources at their own pace. One month later, they completed the post-intervention questionnaire.
- Mixed approach: G3 attended a 3-hour webinar aimed at exploring the e-book content in depth. One week later, students interacted with the e-platform and the three interactive learning modules. They were also introduced to the mobile app, after which they were free to use it at their own pace. One week after that, students completed the post-intervention questionnaire.

**Figure 1:**
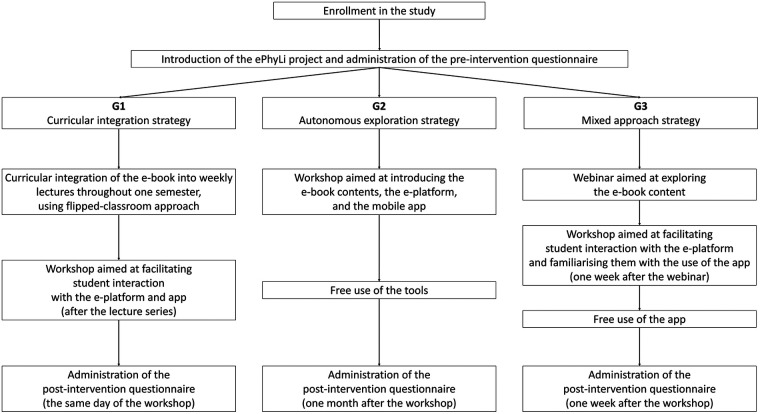
Study flowchart.

Figure 1 illustrates the study design.

A total of 51 participants (22 from G1, 13 from G2, and 16 from G3) completed the pre- and post-intervention learning assessment items. Meanwhile, 61 participants (30 from G1, 16 from G2, and 15 from G3) agreed to complete the post-intervention items assessing the user experience, system usability, gamification and playfulness of the e-platform and mobile app.

### 2.3 Learning tools

The tools' development followed several phases. Firstly, a teaching and learning pedagogical framework for PL was identified. Secondly, an e-book, a serious digital game mobile app, and an e-platform were designed and created in English. Following this, the materials underwent pilot testing to refine the content before finalization. Finally, they were made available in French, Greek, and Italian. Further information about the pedagogical framework and the ePhyLi tools is reported in the Supplementary Material.

#### 2.3.1 E-book

Organized in eight modules, the e-book serves as a comprehensive guide through the roots, concept, and impact of PL on users' lives. It aims to explore the concept of PL in greater depth, to highlight the importance of PL for health, well-being, and personal development, and to explore how PL can be promoted and evaluated in PE classes.

#### 2.3.2 Mobile app

The serious digital game, available on Android and iOS, is a gamified, educational tool designed to improve users' understanding of PL. It features a user-friendly onboarding process, including avatar creation and customizable profiles. A virtual guide named “Buddy” provides in-game orientation and tutorials, reinforcing user engagement. The app's interface includes an interactive list of sequential challenges, a glossary for conceptual support, and a system of badges (awards) to incentivize engagement. The app supports knowledge acquisition and attitudinal development by embedding PL concepts within 13 game-based challenges.

#### 2.3.3 E-learning platform

Through the e-platform, users can read about the ePhyLi project, read and download the e-book, download the app and interact with other digital resources, including two comic e-books and three engaging learning units. For the purpose of the present study, only the interactive module features are described. The three interactive learning modules were designed to enhance understanding through active participation, fostering learners' motivation, confidence, and physical competence. Each module presents realistic school-based scenarios in which students must select appropriate strategies to promote PL within a PE lesson, challenge students at the right level, support holistic assessment practices, and encourage reflection on student growth across the four PL domains: physical, cognitive, affective, and social.

### 2.4 Measures

#### 2.4.1 Learning assessment

To assess participants' knowledge and understanding of PL acquired through the tools, an *ad hoc* questionnaire was integrated as a self-assessment tool at the end of each e-book module. Its primary educational aim was to help participants reflect on and evaluate the specific knowledge they gained. To ensure content validity and pedagogical alignment, the instrument was reviewed and approved by a group of experts in PL. The questionnaire consisted of 20 multiple-choice questions, which included four possible answers, with a single correct option (see Supplementary Material). The questions were organized into four domains: foundational PL concepts; personal and environmental influences; assessment and charting progress; and the role of play, enabling environments, support, and encouragement. The items explored the core principles of PL, fundamentals and theoretical principles, the distinctions between PL and PE, developmental models and frameworks, assessment and teaching strategies, and practical application in PE. Participants completed the questionnaire twice: once prior to using the tools to establish a baseline, and again after engaging with the e-book, e-platform, and mobile app to measure shifts in knowledge and awareness.

#### 2.4.2 Users' experiences with usability and satisfaction in using the e-platform and mobile app

The post-intervention questionnaire package also included questions to evaluate the usability, user experience, usefulness, and gamification elements of the e-platform and the mobile app.

The User Experience Questionnaire short version (UEQ-S) was administered to investigate user experience (10). It consists of 8 items and measures two key dimensions of user experience: *pragmatic quality* (4 items) and *hedonic quality* (4 items). Each item is presented as a semantic differential with opposing terms, rated on a 7-point Likert scale ranging from −3 (horribly bad) to +3 (extremely good). Values <−0.8 represent a negative evaluation, between −0.8 and 0.8 represent a neutral evaluation, and values >0.8 represent a positive evaluation. The UEQ-S was completed twice, once for the e-platform and once for the mobile app.

The System Usability Scale (SUS) was administered to assess the *perceived usability* of the tools (11). It consists of a 10-item questionnaire with statements rated on a 5-point Likert scale ranging from 1 (strongly disagree) to 5 (strongly agree). The items alternate between positive and negative phrasing to reduce response bias. SUS scores are calculated by converting individual item responses into a composite score ranging from 0 to 100, where higher scores indicate better usability. A SUS score of 68 is generally considered an average benchmark for usability. The SUS was completed twice, once for the e-platform and once for the mobile app.

The App Usability Evaluation Questionnaire was administered to investigate the level of *overall satisfaction*, *complexity of use* (ease of use), *learnability* (ease of learning), *intention to use* the application in the future (future use), and *willingness to recommend* it to others (recommended to others) (12). It consists of 5 questions rated using a 5-point scale, with response scales ranging from 1 (strongly disagree) to 5 (strongly agree). This questionnaire was completed once, only for the mobile app.

The Gameful Experience Questionnaire (GAMEFULQUEST) is a self-report instrument designed to measure the perceived gamefulness of system use, capturing users' gameful experience while interacting with gamified environments, such as games, gamified services, or simulators (13). It assesses seven core dimensions of gameful experience: *accomplishment* (the sense of progress and goal achievement), *challenge* (the perception of effort and skill testing), *competition* (experiencing rivalry), *guided* (feeling directed or supported), *immersion* (being absorbed in the experience), *playfulness* (enjoyment and fun), and *social experience* (interaction with others). A 7-point Likert-type scale was used, ranging from 1 (strongly disagree) to 7 (strongly agree). In the present study, only the dimension of playfulness (i.e., the ten questions) was used. The playfulness dimension was completed once, only for the mobile app.

In addition, nine single-ended questions were included in the post-intervention questionnaire package to capture elements of students' perceived usefulness, understanding, and gamification of the e-platform and the mobile app (see Supplementary Material).

#### 2.4.3 Statistical analyses

Data were analyzed using descriptive (mean, standard deviation, percentages) and inferential statistics. More specifically, McNemar's tests were used to analyze the overall difference in percentages between the pre- and post-intervention results. *T*-tests for paired samples were used to analyze differences in the total sample’s pre- and post-intervention scores. Additionally, possible differences in the outcome evaluation of the program across the three groups were examined with analysis of variance (ANOVA) tests for user experience, system usability, gamification, and the playfulness of the e-platform and mobile app, as well as the score derived from the nine single-ended questions. Repeated measures ANOVA was used to analyze differences in learning assessment of knowledge between the pre- and post-intervention in the three participating institutions. ANOVA analyses were followed by *post-hoc* comparisons with Bonferroni adjustment when statistically significant differences were observed. The internal consistency of the various instruments was assessed by Cronbach's *α* coefficients. The significance level was set to *p* < 0.05. The statistical analysis was conducted using IBM SPSS version 29.0 (IBM SPSS Corp., Armonk, NY, USA).

## 3 Results

### 3.1 Pre- and post-intervention learning assessment

The results of the McNemar's test and *t*-test for the pre- and post-intervention learning assessment items (Table 1) showed several key findings across the evaluated domains.

**Table 1:** Learning assessment results.

Items	Pre-intervention	Post-intervention	McNemar *Z*/*t*-test	*p*
Foundational physical literacy concepts				
1. What does physical literacy emphasize?	43 (84.3%)	49 (96.1%)	2.449	**0** **.** **014**
2. Why is experience considered more important than the activity itself in physical literacy?	34 (66.7%)	44 (86.3%)	2.673	**0** **.** **008**
3. How can physical literacy influence behavior towards physical activity?	46 (90.2%)	47 (92.2%)	0.378	0.705
4. How does physical literacy differ from physical education?	39 (76.5%)	45 (88.2%)	2.121	**0** **.** **034**
5. What aspect of physical literacy can potentially contribute to a lifelong engagement with physical activity?	41 (80.4%)	46 (90.2%)	1.667	0.096
Total score	3.98 ± 1.35	4.53 ± 1.10	3.503	**<0** **.** **001**
Personal and environmental influences				
6. Why is there a need for a focus on physical literacy?	44 (86.3%)	43 (84.3%)	0.302	0.382
7. What are the three main philosophies that physical literacy is rooted in?	6 (11.8%)	22 (43.1%)	3.578	**<0** **.** **001**
8. In order of impact, what are the environmental influences on each individual's physical literacy?	7 (13.7%)	14 (27.5%)	1.698	**0** **.** **045**
9. Jurbala's spiral of physical literacy development includes:	28 (54.9%)	39 (76.5%)	2.524	**0** **.** **006**
10. The enhanced physical literacy cycle is:	8 (15.7%)	20 (39.2%)	2.683	**0** **.** **004**
Total score	1.82 ± 1.35	2.71 ± 1.35	4.427	**<0** **.** **001**
Assessment and charting progress				
11. Physical education is a multi-dimensional concept and as such, teachers should consider progress in relation to which aspects?	32 (62.7%)	42 (82.4%)	2.500	**0** **.** **006**
12. What key principles should we focus on when charting or assessing progress in relation to physical education and physical literacy?	37 (72.5%)	45 (88.2%)	2.309	**0** **.** **010**
13. An Authentic Core Task linked to physical education and physical literacy is:	34 (66.7%)	39 (76.5%)	1.387	0.083
14. SOLO Taxonomy has a progression through:	23 (45.1%)	30 (58.8%)	1.528	0.063
15. What four physical literacy aspects would we like to see from students in physical education?	32 (62.7%)	34 (66.7%)	0.535	0.296
Total score	3.10 ± 1.54	3.73 ± 1.47	3.108	**0** **.** **003**
The role of play, enabling environments, support and encouragement				
16. Physical literacy within physical education should focus on:	31 (60.8%)	38 (74.5%)	1.528	0.063
17. What are the best approaches to working with early years children in physical education?	29 (56.9%)	37 (72.5%)	1.789	**0** **.** **037**
18. Enabling environments for physical education should include:	29 (56.9%)	34 (66.7%)	1.091	0.138
19. For a holistic physical education experience, what sort of play should children have access to?	33 (64.7%)	42 (82.4%)	2.324	**0** **.** **010**
20. Movement forms associated with physical education that provide a breadth of experience should include:	37 (72.5%)	46 (90.2%)	2.714	**0** **.** **003**
Total score	3.12 ± 1.32	3.86 ± 1.31	3.356	**0** **.** **002**

Data are reported as the number of correct answers (percentage) and mean ± standard deviation.

Bold values indicate statistical significance at *p* < 0.05.

Regarding the foundational PL concepts domain (Questions 1–5), participants showed a relatively high baseline knowledge of them. Nevertheless, statistically significant knowledge gains were observed in several items (1, 2 and 4), with a significant improvement in the mean total score (*p* < 0.001). This suggests that participants with strong prior knowledge also benefited from using the tools, particularly regarding the more nuanced or applied aspects of the subject.

The domain of personal and environmental influences (Questions 6–10) showed the most pronounced learning gains. Post-intervention correct responses increased significantly for most items, with the largest gains observed in questions 7 (+31.3%), 9 (+21.6%), and 10 (+23.5%; all *p* < 0.01). The statistically significant increase in the mean total score (*p* < 0.001) highlights the effectiveness of the digital tools in addressing knowledge gaps in these more complex or less familiar content areas.

The domain of assessment and charting progress (Questions 11–15) showed a moderate, but statistically significant improvement in knowledge. While some items showed smaller or non-significant gains, the overall mean score of these questions increased significantly (*p* = 0.003). This suggests that while the tools were effective in reinforcing core implementation concepts, additional experiential or hands-on learning activities may be needed to further enhance understanding in these areas.

Lastly, knowledge relating to the role of play, enabling environments, support, and encouragement domain (Questions 16–20) also showed a statistically significant improvement using the tools (*p* = 0.002). This indicates that the tools were also effective in teaching key practical strategies for the implementation and application of PL concepts.

To control for possible differences in the learning strategies used, repeated measures ANOVA was performed. The analysis (after sphericity was assumed; Mauchley's *W* = 0.893, *p* = 0.380) revealed that there were statistically significant differences for the interaction between Time and Groups [*F*_(2,48)_ = 9.225, *p* < 0.001, *η*^2^ = 0.278], while no significant differences were observed for the overall interaction between Time, Groups and Modules [*F*_(6,144)_ = 1.665, *p* = 0.134, *η*^2^ = 0.065]. *Post-hoc* comparisons showed that the strategies used for G1 contributed most to the overall improvement in knowledge results, as the students in this group statistically significantly improved their PL knowledge (grand M pre = 2.97, grand M post = 4.25, *p* < 0.001). This was not the case in the two other groups (G2: grand M pre = 2.56, grand M post = 2.73, *p* = 0.477; G3: grand M pre = 3.42, grand M post = 3.75, *p* = 0.129). Even though they marginally improved their knowledge, this change was not statistically significant. Table 2 shows the learning results of each institution.

**Table 2:** Learning assessment results stratified by groups.

Domains	Pre-intervention	Post-intervention
G1		
Foundational PL concepts	3.91 ± 1.31	4.73 ± 0.77
Personal and environmental influences	2.00 ± 1.11	3.36 ± 1.36
Assessment and charting progress	3.00 ± 1.57	4.32 ± 1.17
The role of play, enabling environments, support and encouragement	2.95 ± 1.25	4.59 ± 0.96
Grand M	2.97 ± 3.31	4.25 ± 1.01
G2		
Foundational PL concepts	3.62 ± 1.81	3.85 ± 1.77
Personal and environmental influences	1.46 ± 0.97	1.54 ± 0.78
Assessment and charting progress	2.23 ± 1.59	2.46 ± 1.76
The role of play, enabling environments, support and encouragement	2.92 ± 1.38	3.08 ± 1.50
Grand M	2.56 ± 1.44	2.73 ± 1.45
G3		
Foundational PL concepts	4.38 ± 0.89	4.81 ± 0.40
Personal and environmental influences	1.88 ± 0.62	2.75 ± 1.07
Assessment and charting progress	3.94 ± 1.00	3.94 ± 0.93
The role of play, enabling environments, support and encouragement	3.50 ± 1.37	3.50 ± 1.10
Grand M	3.42 ± 0.97	3.75 ± 0.88

Data are reported as mean ± standard deviation.

### 3.2 User experience, system usability, gamification, and playfulness of the e-platform and mobile app

#### 3.2.1 User experience

Table 3 shows the results of the user experience evaluations of the e-platform and app. Both tools showed excellent internal consistency, with Cronbach's *α* coefficients exceeding 0.90 for all UEQ-S dimensions. The UEQ-S revealed moderate positive evaluations for both tools. According to UEQ-S interpretation guidelines, both the pragmatic quality and the hedonic quality dimensions demonstrated acceptable user experience levels for both tools. The overall user experience of the app showed no statistically significant difference between the three groups [*F*_(2,60)_ = 0.588, *p* = 0.559, *η*^2^ = 0.020]; while a significant difference was observed for the e-platform [*F*_(2,60)_ = 3.207, *p* = 0.048, *η*^2^ = 0.100]. The Tukey *post-hoc* comparison revealed that G2 (1.26 ± 1.36) rated significantly higher the overall quality of the e-platform (*p* = 0.038) than G1 (0.28 ± 1.35). No further statistically significant differences were observed.

**Table 3:** User experience and system usability evaluations of the e-platform and app.

Dimensions	Score	Min-Max	Cronbach's *α*
UEQ-S e-platform			
Pragmatic quality	0.72 ± 1.40	−3 to 3	0.91
Hedonic quality	0.52 ± 1.39	−3 to 3	0.90
Overall quality	0.62 ± 1.29	−3 to 3	0.90
UEQ-S game app			
Pragmatic quality	0.78 ± 1.43	−3 to 3	0.94
Hedonic quality	0.43 ± 1.40	−3 to 3	0.94
Overall quality	0.61 ± 1.36	−3 to 3	0.94

Data are reported as mean ± standard deviation.

#### 3.2.2 System usability

The e-learning platform achieved a SUS score of 63.49 ± 13.86 (range: 40–98; Cronbach's *α* = 0.73). The mobile app scored slightly lower at 60.67 ± 16.2 (range: 10–95; Cronbach's *α* = 0.78), indicating room for usability improvements. Both scores fall slightly below the commonly cited usability benchmark of 68 for acceptable usability, indicating marginal usability issues requiring further attention. The system usability for the platform did not reveal any statistically significant differences between the three groups [*F*_(2,59)_ = 1.638, *p* = 0.204, *η*^2^ = 0.056]. However, significant differences were observed for the app [*F*_(2,59)_ = 5.411, *p* = 0.007, *η*^2^ = 0.160]. The Tukey *post-hoc* comparison revealed that G2 (68.91 ± 17.13) rated significantly higher than G3 (50.71 ± 9.63) in the system usability of the app (*p* = 0.005).

#### 3.2.3 App usability and playfulness

Table 4 shows the app usability and playfulness results.

**Table 4:** App usability and playfulness results.

Scales	Score	Min-Max	Cronbach's *α*
App usability	18.56 ± 4.20	10–25	0.92
Overall satisfaction	3.67 ± 0.96	2–5	
Ease of use	3.82 ± 1.06	2–5	
Ease of learning	3.74 ± 0.91	2–5	
Future use	3.61 ± 0.94	2–5	
Recommended to others	3.72 ± 0.95	2–5	
App playfulness	36.21 ± 8.32	21–50	0.96

Data are reported as mean ± standard deviation.

The dedicated app usability questionnaire yielded an overall score of 18.56 ± 4.20 out of 25 (range: 10–25) with excellent reliability (Cronbach's *α* = 0.92). Individual component analysis revealed that ease of use received the highest rating, followed by ease of learning and recommendation to others. App usability scores suggest generally positive attitudes toward the app, particularly in terms of usability and perceived utility. For the institutional comparisons, the only statistically significant difference was estimated for the overall satisfaction variable [*F*_(2,60)_ = 4.684, *p* = 0.013, *η*^2^ = 0.139]. The *post-hoc* comparison showed that G3 (3.07 ± 0.80) perceived significantly lower overall satisfaction from the app use compared to G1 (3.80 ± 0.89; *p* = 0.035) and G2 (4.00 ± 1.03; *p* = 0.016). No further significant differences were observed for the remaining variables.

The playfulness dimension of the GAMEFULQUEST questionnaire, focusing on enjoyment and engagement, demonstrated strong results. This measure showed exceptional internal consistency (Cronbach's *α* = 0.96), indicating that participants perceived the gamification elements as engaging and motivating. The comparison of app playfulness between the three groups was statistically significant [*F*_(2,60)_ = 4.024, *p* = 0.023, *η*^2^ = 0.122]. The only statistically significant difference was the higher evaluation of playfulness from the G2 (39.4 ± 9.59) compared to G3 (31.53 ± 3.85; *p* = 0.020).

### 3.3 Perceived learning and confidence in applying physical literacy

Results about the participants’ perceived learning and confidence in applying PL concepts, after using the ePhyLi e-book, mobile app, and e-platform, showed a strong positive impact of the ePhyLi digital tools on them. Referring to the learning from the mobile app, the average score for learning something new about PL was 3.45 ± 0.97 (out of 5). This suggests that the majority of participants felt that the game was effective in introducing new concepts and enhancing their understanding of PL. Similarly, the mean score for learning from the e-platform was 3.55 ± 1.01 (out of 5), reflecting an average-to-high level of perceived knowledge gain from engaging with the e-platform's digital content. Referring to the confidence in applying PL concepts to academic or professional practice, the mean score was 3.53 ± 0.90. Similar to the knowledge acquisition scores, this value still indicates that most participants felt adequately prepared to transfer their learning into practical settings. No statistically significant differences were observed between the students from the three institutions (*p* > 0.05), revealing that all students showed an almost similar level of perceived learning and confidence in applying PL concepts.

### 3.4 Perceived educational value and usefulness

Participants rated both the e-platform and the mobile app favorably in terms of educational utility (Table 5). Distribution analysis showed that 56.7% of participants rated the e-platform as considerably to extremely useful in understanding the PL concept, while 55.8% provided similar ratings for the mobile app. When compared to traditional learning methods, both tools demonstrated moderate effectiveness in improving user PL concept understanding. Notably, 50% of participants agreed that the e-platform improved their understanding, while 42.6% expressed similar sentiments regarding the mobile app. The gamification elements received positive evaluation (3.66 ± 1.08), with 55.7% of participants agreeing that gamified approaches helped reinforce key concepts better than traditional methods. Additionally, 52.4% of participants reported that gamification elements enhanced their motivation to engage with content (3.57 ± 0.97).

**Table 5:** Perceived educational value and usefulness of the tools.

Educational utility	e-Platform	App
Effectiveness in understanding the PL concept	3.67 ± 0.95	3.59 ± 1.01
Effectiveness in improving user understanding compared to traditional learning methods	3.47 ± 0.66	3.43 ± 1.01

Data are reported as mean ± standard deviation.

The Tukey follow-up comparisons revealed that, in general, G3 rated significantly lower than the other two groups on all questions for the perceived educational value and usefulness of the app and platform (*p* < 0.05), except for the use of the e-learning platform compared to more traditional learning approaches (*p* > 0.05). G1 and G2 held almost similar perceptions.

## 4 Discussion

Findings demonstrate that participants' understanding of PL was significantly improved by the ePhyLi tools, even among those participants with prior knowledge. The tools were also effective at addressing knowledge gaps, particularly about more nuanced or applied PL aspects and complex or less familiar content areas. The most significant learning gains were observed in topics related to the personal and environmental influences on the individual PL journey. The results of the three-strategy comparison showed that the most effective way to improve participants' PL knowledge was to integrate the e-book into weekly lectures throughout a semester using a flipped-classroom approach, alongside the use of the e-platform and app. This highlights that the long-term curricular integration of digital learning resources is more effective than autonomous study or a mixed method.

Most participants felt that the mobile app and the e-platform were effective in introducing new concepts and deepening their understanding of PL. Furthermore, the gamification elements were perceived as engaging and motivating, which helped to reinforce the acquisition of key concepts. Participants also reported a strong sense of perceived learning and increased confidence in applying PL concepts to their academic or professional practices. This is consistent with scientific evidence demonstrating that digital learning tools can enhance learning outcomes (14, 15).

By contrast, knowledge gains in PL assessment and monitoring were moderate, likely to reflect the practical nature of this domain, which may require additional experiential or hands-on learning activities. This finding also points to a potential area for future tool developments, suggesting that the digital format may need to be enhanced with interactive simulations or guided practical exercises to support learning in this area fully. For instance, a specific pedagogical framework could involve longitudinal branching simulations. In these scenarios, users are presented with dynamic profiles of students tracked over an extended timeframe. Participants would be required to assess PL indicators at multiple time points, make strategic pedagogical decisions, and observe the simulated long-term consequences of those choices on the students' PL journeys. This approach would bridge the gap between static theory and the dynamic, long-term nature of PL assessment and monitoring. It is also important to note that, despite improvements in the evidence base for PL assessment and analysis (16–19), gaps remain, particularly in the assessment of cognitive and affective domains (20). As teachers play a crucial role in promoting children's well-being and holistic development at school, digital tools that support teachers' and other educators' learning could improve the quality of education (21).

The results also provided valuable insights into users' experience with the e-learning platform and mobile app. Overall, participants gave the tools a slightly positive user-experience rating, especially in terms of pragmatic value. However, SUS scores for both the e-platform and the app fell below the conventional benchmark of 68 (22), indicating that usability was acceptable but still in need of refinement. This suggests that, while the digital tools demonstrate an educational utility, further improvements to the interface and interaction flow are warranted. The app usability evaluation showed that participants found the mobile app easy to use and learn, with high scores for willingness to recommend and future use intention. The playfulness dimension results indicated that the gamification elements were engaging and motivating, effectively enhancing user interaction and enjoyment. Gamification is receiving high interest for academic learning (23), showing that gamified educational interventions yield substantial improvements in learning outcomes and motivation (24, 25). As demonstrated by Baah et al. (15), gamification supports learning by reducing the mental effort needed to study and helping students memorize concepts more effectively. It must also be noted that primary education student-teachers (G3) showed lower app usability and playfulness when using the app. They also had a lower perception of the educational value and usefulness of the e-platform and app compared to the other two groups. Due to the lack of data explaining these results, we can only hypothesize that this discrepancy may be related to two main factors. On the one hand, it may be related to differences in curricula and professional identities. Sport Science students (G1 and G2), whose programs are strongly focused on PA and health, are more likely to view these tools as directly relevant to their future careers, whereas primary education student-teachers (G3), enrolled in a generalist program where PE is only one subject among many, may not consider them a priority. On the other hand, international literature (26) indicates that engagement is higher when digital tools closely match users' roles, expectations, and professional context. Accordingly, some gamification and user interface/user experience elements may have seemed less relevant to the interdisciplinary demands of primary school teaching, negatively affecting G3's perceptions of usability, playfulness, and educational value. Given these findings, future design solutions should place greater emphasis on interdisciplinary connections. Highlighting how PL can be integrated with other primary school subjects could increase its perceived value and relevance for G3 participants. Nevertheless, these interpretations remain speculative and should be examined in future qualitative and mixed-methods studies.

The present study showed significant strengths. Initially, a robust approach was used to develop and evaluate digital learning tools for PL, using innovative, interactive, gamified, and well-structured content to foster engagement, motivation, and a deeper understanding of PL concepts. Finally, a diverse range of accessible digital learning tools, including an e-book, a serious digital game mobile app (available on Android and iOS), and an e-learning platform with interactive learning units, and two comic e-books, were developed and are available in different languages (English, Greek, Italian, and French). Some limitations were also identified. Although the total sample size could be considered adequate for a pilot study, as it exceeded the average of 40 participants found in Schrepp et al.'s study (10), a larger sample size would be necessary to provide stronger findings regarding the effectiveness of the ePhyLi tools and the generalizability of user experience outcomes. A further limitation is that all evaluation materials were administered in English. Given that all participants were proficient in English, but English was not the first language for everyone, minor issues related to comprehension might have occurred. However, this choice was necessary for methodological standardization, as the sample comprised students from different countries. The use of English for all questionnaires ensured reliable comparability of results between the three groups and that any observed differences in scores were attributable to the learning strategy. Additionally, it is important to note that the differences in intervention conditions (i.e., pedagogical strategies, total duration, and instructional exposure time) reflected the teaching strategies commonly employed in higher education. While these variations were structurally aligned with real-world academic delivery, from a strictly experimental standpoint, they introduce confounding factors. Consequently, it is difficult to determine whether the differences in knowledge gains were solely due to the specific instructional strategy, the total exposure time, the intensity, or the timing of the assessments. To address this, future research, with similar exposure periods and synchronized assessment schedules, could be performed. Another limitation of this study concerns the learning assessment tool. The questionnaire was developed as a component of the e-book, functioning as a chapter-by-chapter self-analysis tool rather than a standalone learning evaluation scale. While its content validity was rigorously ensured through an expert panel to guarantee strict alignment with the knowledge presented in each module, formal statistical validity and reliability testing were not conducted. Consequently, while effective for its embedded pedagogical purpose, the tool lacks independent statistical standardization, which should be a focus for future research seeking to adapt these items into a standardized evaluation instrument.

## 5 Conclusion

The ePhyLi digital tools demonstrated significant potential as innovative resources for fostering PL among university student-teachers. Pilot testing results confirmed that they effectively enhanced students' knowledge, confidence, and engagement, providing an interactive and motivating learning environment. To improve students' PL knowledge, it is necessary to integrate the e-book into weekly lectures throughout a semester using a flipped-classroom approach alongside the e-platform and app. Future development could focus on refining usability, incorporating experiential learning elements, and leveraging gamification strategically to maximize educational impact. Taking these factors into account, the ePhyLi digital tools are a valuable and accessible educational technology resource for preparing future teachers. They have substantial potential to promote PL and enhance the quality of education for a wide range of end users. To ensure long-term utility, all ePhyLi tools will remain fully operational and accessible for at least three years post-project, with partners guaranteeing continuous updates through their integration into ongoing higher education and continuous education programs.

## Data Availability

The raw data supporting the conclusions of this article will be made available by the authors, without undue reservation.
