# Supplementary material for: ePhyLi digital tools improve student-teachers' physical literacy knowledge: evaluation of usability, user experience, and learning outcomes
Source: Front Sports Act Living. 2026 Jul 22;8:1875835. doi: 10.3389/fspor.2026.1875835 (PMC13437456; doi:10.3389/fspor.2026.1875835)
Supplement: Supplementary file 1 [file Datasheet1.pdf]

frontiers

Supplementary Material

1 Pedagogical framework

Desk and field research were conducted to develop the pedagogical framework.
Desk research was conducted on European and international priorities in PL and connections to PE, including PE teaching approaches and the assessment of PL in PE. The role of gamification elements in innovative educational strategies was also investigated to reflect on the current state of the art, to examine educational priorities at international, European and national levels and to design innovative approaches. Theories and pedagogical approaches were also considered, including the Self-Determination Theory[1], the Constraints-Led Approach[2], and the Models-Based Practice[3]. An investigation was conducted into the historical development, main trends and current challenges in the field of PE and the integration of PL in PE curriculum, with national reports provided by project partners.
An online survey was conducted with 92 university students and PE or generalist teachers across four European countries (Cyprus, the Czech Republic, Italy, and Serbia) to explore their understanding of PL and its impact on sport and PA engagement. Focus group sessions involving 89 participants were also organized in Cyprus, the Czech Republic, and Serbia to document the prior knowledge of PL held by students and teachers, and to identify ways to cultivate PL through physical education.
The framework guided the design of a gamified digital learning approach towards the development of an e-book, a serious digital game (mobile app), and an e-platform with interactive learning materials and two comic e-books.

2 E-book

The e-book comprises eight modules. It introduces and deepens the understanding of PL, beginning with its philosophical foundations and global perspectives (Module 1), and highlights the importance of PL for health, well-being, and personal development, emphasizing the influence of both personal experiences and social factors on PA (Module 2). Assessment and progress tracking in PL and PE are explored using authentic core tasks and effective monitoring strategies (Module 3). The e-book outlines approaches for fostering positive PA experiences in early childhood (Module 4) and among learners aged 5-18 through thoughtful curriculum design and extracurricular planning (Module 5). It also presents effective teaching strategies and pedagogical models that support the development of PL (Modules 6 and 7). Finally, the e-book underscores the importance of inclusive practices to ensure the holistic development of all children (Module 8). Each module includes reflective exercises designed to encourage discussion and engagement with the topic, as well as self-assessment questions intended to

---

[1] Renshaw, I., Oldham, A. R., & Bawden, M. (2012). Nonlinear Pedagogy Underpins Intrinsic Motivation in Sports Coaching. The Open Sports Sciences Journal, 5(1), 88–99. https://doi.org/10.2174/1875399X01205010088

[2] Ryan, R. M., & Deci, E. L. (2018). Self-determination theory: Basic psychological needs in motivation, development, and wellness (Paperback edition). The Guilford Press. ISBN:

1462538967

[3] Casey, A., & MacPhail, A. (2018). Adopting a models-based approach to teaching physical education. Physical Education and Sport Pedagogy, 23(3), 294–310. https://doi.org/10.1080/17408989.2018.1429588

ensure a thorough understanding of the subject matter. Before its use in this study, the e-book underwent a preliminary piloting phase to assess the clarity of its contents and its practical utility for the target audience. Based on the feedback received, minor adjustments were made to the tool to ensure it was well-structured and easy to comprehend.

3 Mobile app

The 13 game-based challenges provide activities designed to improve understanding of how PL can be promoted in both school-based and real-life scenarios. Challenges also require players to apply their knowledge of PE pedagogy, adapt to contextual constraints (e.g., class characteristics, equipment availability), and make informed instructional choices. They also draw on evidence-informed educational strategies and pedagogical models (e.g., Mosston's spectrum of teaching styles and models like Sport Education, Cooperative Learning, and Teaching Personal and Social Responsibility) to foster reflective and inclusive practice in PE. For instance, one scenario illustrates a sixth-grade educational gymnastics lesson, where players must select suitable teaching styles, adapt activities for inclusivity, prioritise pedagogical goals (e.g., safety, enjoyment), and apply appropriate assessment strategies.

4 E-platform

The e-platform also gives the possibility to read two comic e-books. They illustrate the journeys of Roly and Silvana in embracing PA and overcoming challenges. The comic e-books aim to make learning about PL engaging and relatable, contributing to the digital transformation of university curricula.

4.1 Roly's comic e-book

It tells the story of Roly, a boy whose life has been deeply shaped by his love for PA, camaraderie, and competition. From his early years of carefree play in the local park to learning new sports in the Army, Roly's journey is one of resilience, friendship, and lifelong learning through sports. Despite a rigorous work schedule, he always found time for his passions, such as cycling, rugby, dance, and more. As he grew older, Roly continued to explore new sports and inspire those around him, especially his children. His journey captures the joy of an active life and the powerful impact of sports on confidence, connection, and well-being.

4.2 Silvana's comic e-book

It tells the story of Silvana, a girl who grew up in a bustling city with limited open spaces yet discovered a love for PA through her school's extracurricular activities. Despite challenges with body image and confidence, Silvana's experiences in sports like volleyball, skating, and fencing became her path to self-expression and resilience. Through the mentorship of supportive teachers, she discovered that PA was not only a source of health but also a way to connect with others. As an adult, she became a passionate PE teacher and community advocate, promoting PL for all. Silvana's journey reflects the transformative power of sports in building confidence, personal growth, and lifelong health.

5 Physical Literacy knowledge questionnaire

The learning evaluation was carried out by administering a questionnaire before and after the intervention, involving the following questions. The correct answer is in bold.

1. What does physical literacy emphasize?
    a. Competitiveness in sports.
    b. Physical strength and endurance.
    c. Holistic integration of physical, cognitive, and affective domains.
    d. Mastery of specific sports.
2. Why is experience considered more important than the activity itself in physical literacy?
    a. It fosters a competitive spirit.
    b. It ensures compliance with rules.
    c. It enhances skill development.
    d. **It contributes to meaningful engagement and personal growth**.
3. How can physical literacy influence behavior towards physical activity?
    a. By imposing strict fitness regimes.
    b. **By cultivating an intrinsic motivation to engage in physical activity.**
    c. By mandating participation in a variety of sports.
    d. By prioritizing performance outcomes.
4. How does physical literacy differ from physical education?
    a. Physical literacy is only about skill development.
    b. **Physical literacy incorporates a broader understanding of movement and its value.**
    c. Physical education does not involve physical activity.
    d. Physical literacy is less structured than physical education.
5. What aspect of physical literacy can potentially contribute to a lifelong engagement with physical activity?
    a. **Development of motivation and confidence in one's ability to be active.**
    b. Mandatory daily exercises.
    c. Emphasis on performance metrics.
    d. Focus on team sports.
6. Why is there a need for a focus on physical literacy?
    a. **To enrich life, enhance well-being, provide breadth and balance to life experiences and to realize our self-esteem.**
    b. To become fitter, stronger, more flexible, and have better endurance.
    c. So that we can write more clearly about our physical activity experiences.
    d. So that we can become better at fundamental movement skills, which will help us become better athletes.
7. What are the three main philosophies that physical literacy is rooted in?
    a. Idealism, realism and pragmatism.
    b. Existentialism, progressivism and constructivism.
    c. **Monism, existentialism and phenomenology.**
    d. Behaviorism, humanism and social reconstructionism.
8. In order of impact, what are the environmental influences on each individual's physical literacy?
    a. Policy, social, organizational, culture and physical.
    b. **Culture, policy, physical, organizational and social.**
    c. Physical, social, policy, culture and organizational.
    d. Culture, social, policy, organizational and physical.

9. Jurbala’s spiral of physical literacy development includes:
    a. Relaxing, training hard, increasing fitness and enriching the quality of life.
    b. Specializing in one activity, increasing physical fitness, increasing participation and enriching the quality of life.
    c. Improving fundamental movement skills, increasing self-esteem, becoming less sedentary, and enriching the quality of life.
    d. **Improving movement through participation, increasing self-efficacy, engaging in different activities and enriching the quality of life.**
10. The enhanced physical literacy cycle is:
    a. **Competence-confidence; motivation-active participation; fun-enjoyment-pleasure; connectedness-relatedness; and creativity-agency.**
    b. Motivation-active participation; fun-enjoyment-pleasure; connectedness-relatedness; creativity-agency; and competence-confidence.
    c. Fun-enjoyment-pleasure; competence-confidence; motivation-active participation; connectedness-relatedness; and creativity-agency.
    d. Creativity-agency; competence-confidence; motivation-active participation; fun-enjoyment-pleasure; and connectedness-relatedness.
11. Physical education is a multi-dimensional concept and as such, teachers should consider progress in relation to which aspects?
    a. **Physical, affective, cognitive and social.**
    b. Speed, agility, endurance and strength.
    c. Fitness, health, agility and balance.
    d. Flexibility, power, muscular endurance and skill.
12. What key principles should we focus on when charting or assessing progress in relation to physical education and physical literacy?
    a. Focus on what sports the children might excel at in the future.
    b. Focus on comparing progress in relation to their peers and grading them.
    c. Focus on targets set by the teacher.
    d. **Focus on the individual and their holistic development over time.**
13. An Authentic Core Task linked to physical education and physical literacy is:
    a. A challenge that children must attain within a particular activity.
    b. **A challenge that allows children to show what they can do, feel and know about an activity.**
    c. A challenge that focuses on core fitness within an activity.
    d. A challenge that is similar to other tasks that they have done in the past.
14. SOLO Taxonomy has a progression through:
    a. Post Structural, Structural, Extended, Advanced, Excellent.
    b. Unistructural, Relational, Multistructural, Abstract, Extended Abstract.
    c. **Prestructural, Unistructural, Multistructural, Relational, Extended Abstract.**
    d. Beginner, Improver, OK, Advanced, Excellent
15. What four physical literacy aspects would we like to see from students in physical education?
    a. **Engaged, confident, physically competent and knowledgeable.**
    b. Knowledgeable, motivated, excellent, social.
    c. Well-behaved, active, good at one sport, friendly.
    d. Literate, expert, well-behaved and good attendance.

16. Physical literacy within physical education should focus on:
    a. **Holistic development, providing a range of movement experiences, being inclusive and personalized.**
    b. Holistic development, improving agility, endurance and strength.
    c. Providing a range of movement experiences, improving fitness, health and balance.
    d. Being inclusive and personalized, improving flexibility, power, and muscular endurance.
17. What are the best approaches to working with early years children in physical education?
    a. Fitness-focused, play-based, varied and inclusive.
    b. Competitive, play-based, progressive and varied.
    c. Always outdoors, varied, inclusive and focused on fitness.
    d. **Play-based, progressive, varied, and inclusive.**
18. Enabling environments for physical education should include:
    a. Support, competition and challenge.
    b. Agility, balance and coordination.
    c. **Variety, challenge and support.**
    d. Fitness, skill development and strength training.
19. For a holistic physical education experience, what sort of play should children have access to?
    a. **Object, expressive movement, exercise and outdoor play.**
    b. Unistructural, relational, multistructural, abstract play.
    c. Online, physical, cooperative and social play.
    d. Independent, solitary, role and social play.
20. Movement forms associated with physical education that provide a breadth of experience should include:
    a. Football, basketball, handball, hockey, tennis and cricket.
    b. Fitness, dance, badminton, athletics, gymnastics and ballet.
    c. Games, athletics, rugby, football, skiing and ice skating.
    d. **Games, athletics, martial arts, expressive and aesthetic, adventure and health and fitness.**

6 Nine single-ended questions

1. To what extent do you feel you have learned something new about physical literacy after using the game?
    - Five-point scale: “not at all” to “a great deal”.
2. To what extent do you feel you have learned something new about physical literacy after using the e-platform?
    - Five-point scale: “not at all” to “a great deal”.
3. To what extent do you feel confident in applying what you have learned to your academic or professional practice?
    - Five-point scale: “not at all” to “a great deal”.
4. How useful was the game in helping you understand the concept of physical literacy?
    - Five-point scale: “not useful at all” to “extremely useful”.
5. How useful was the e-learning platform in supporting your learning about physical literacy?
    - Five-point scale: “not useful at all” to “extremely useful”.
6. Using the game improved my understanding compared to traditional learning methods.
    - Five-point scale: “strongly disagree” to “strongly agree”.
7. Using the e-learning platform improved my understanding compared to traditional learning methods.
    - Five-point scale: “strongly disagree” to “strongly agree”.
8. Compared to traditional learning approaches, gamification helped reinforce key concepts better.
    - Five-point scale: “strongly disagree” to “strongly agree”.
9. Did the gamification elements of the game (e.g., challenges, rewards, interactivity) enhance your motivation to engage with the content?
    - Five-point scale: “not at all” to “a great deal”.
